# Editorial: Small animal imaging: Technological and methodological advances to improve the translational power

**DOI:** 10.3389/fmed.2022.1099233

**Published:** 2022-12-12

**Authors:** Monique R. Bernsen, Wendy McDougald, Laura Mezzanotte, Carmel M. Moran, Adriana Tavares, Louise van der Weerd

**Affiliations:** ^1^AMIE Core Facility, Erasmus MC, University Medical Center Rotterdam, Rotterdam, Netherlands; ^2^Siemens, Molecular Imaging, Hoffman Estates, IL, United States; ^3^BHF-University Centre for Cardiovascular Science, University of Edinburgh, Edinburgh, United Kingdom; ^4^Department of Radiology and Nuclear Medicine, Erasmus MC, University Medical Center Rotterdam, Rotterdam, Netherlands; ^5^Department of Human Genetics, Leiden University Medical Center (LUMC), Leiden, Netherlands; ^6^Department of Radiology, Leiden University Medical Center (LUMC), Leiden, Netherlands

**Keywords:** small animal imaging, standardization, technological advances, methodological advances, translational power, harmonization

Small-animal imaging is a dynamic research field with continuing technological and methodological advances. It plays an important role in biomedical research by helping us to increase our understanding of biological and disease processes, to evaluate new drugs and treatments, and to identify and evaluate imaging biomarkers. In this respect it is important to consider the many aspects involved in the planning, design, and execution of small animal imaging studies. This includes, amongst others, the choice of animal model, the imaging technique to be used, aspects regarding animal handling and monitoring, data processing and analysis methods and method quantification and calibration. All these aspects are important with regards to adhering to the 3R principles for ethical use of laboratory animals in scientific research, and in strengthening the scientific value and translational power of the obtained results.

This Special Topic aims at generating a widespread understanding of the importance of robust and standardized methods in small animal imaging. This Special Topic contains 12 papers, eight original research manuscripts and four (mini-)reviews, describing various aspects of animal handling, tracer development and imaging techniques.

In the papers by Miranda et al., Proesmans et al., and Ren et al. some specific challenges encountered in small animal brain imaging are identified and methodological advances to overcome the challenges and to improve robustness of research methods are discussed. Proesmans et al. highlight the challenges of inter- and intra-subject variability with the most used quantification methods for glucose metabolism in the brain, and issues with the use of different PET acquisition methods across studies. In their paper they then present a framework for statistical voxel-based analysis of glucose uptake in the rat brain using histogram-based intensity normalization that enables more standardized and harmonized quantification of brain glucose metabolism. A broader approach to the challenges and opportunities in small animal imaging in brain research is presented in the paper by Ren et al.. In their mini-review they discuss recent technological developments in this field including hybrid and multiscale imaging, data processing methods, awake animal imaging, transcriptomics, and on-chip pharmacokinetics. Miranda et al. further zoom in on the confounding effect of anesthesia of laboratory animals during brain imaging and discuss the pros and cons of awake, but restrained, animal imaging and argue for an approach for non-restrained, awake imaging using advanced motion correction methodology. Also in the area of brain imaging, Llambrich et al. report on the added value of a longitudinal, multi-modal imaging approach for multi-system analysis of complex genetic disorders. In a Down syndrome mouse model, they were able to show that such a research approach could reveal the alterations in the coordinated morphogenesis of brain and skull during disease development and response to treatment.

The importance and added value of multi-modal, multiscale and longitudinal imaging approaches are also highlighted in four other papers in this Special Topic. In recent years, the use of zebrafish for disease models has been increasing. Zebrafish have several advantages compared to other vertebrate models used in modeling human diseases, particularly for large-scale genetic mutant and therapeutic compound screenings. Most researchers use the embryonic or larval stage where the zebrafish is fully transparent and can be readily imaged using standard light microscope techniques. To fully utilize the potential of zebrafish for disease models imaging, techniques not limited by the need for transparent subject are needed. In the study by Tucker et al., a new PET/CT imaging platform for adult zebrafish imaging that can maintain normal aquatic physiology during scanning is presented, thereby enabling longitudinal assessment of molecular interactions within the adult zebrafish. Suchacki et al. report on the use of a total-body positron emission tomography (PET) network analysis of murine ^18^F-FDG scans, as a biomarker of glucose metabolism in bones. The reported approach is suitable for studying dynamic multi-bone metabolic interactions *in vivo* and due to the diversity of PET radiotracers alongside the advent of clinical total-body PET systems the technique could be used to reveal new physiological and pathological tissue interactions beyond skeletal metabolism. With regards to bone imaging, Menendez et al., demonstrate the feasibility of dose reduction during ^18^F-Sodium fluoride imaging of osteoblastic activity *via* the use of digital photon counting PET/CT. The need for multi-scale imaging in elucidating complex disease processes is also discussed by Tiwari et al. In their review paper they describe complex mechanisms in cardiovascular disease focusing on changes in endothelial cells lining the blood vessels, and how developments in preclinical multi-scale imaging can aid in enhancing the biological understanding of the interactions between endothelial cells and the immune system. In addition, they argue how this could lead to earlier diagnoses and ways for improved patient management. Rastogi et al. also discuss the importance of multi-modal molecular imaging in cardiovascular disease research. In their paper they focus on aortic aneurysms and highlight the need for better imaging biomarkers for risk-assessment in patients with an aortic aneurysm. They discuss recent developments in molecular imaging techniques and stress that despite the identification of very promising markers and imaging targets, clinical translation is still limited and that in-patient testing and clinical trials of these techniques are needed.

Identification and evaluation of imaging biomarkers and imaging agents are, as mentioned, an important aspect in the field of small animal imaging. In this Research Topic two papers report on improved methods for the production of specific imaging agents. Dai et al. tested a method for more efficient purification of the Vesicular monoamine transporter type 2-targeting tracer, ^18^F-FP-(+)-DTBZ. Change into a commonly used HPLC-based method they propose a modified solid-phase extraction method that significantly simplifies tracer purification and reduces processing time without loss of radiochemical purity or *in vivo* performance. In the field of nanoparticle-based, imaging agents, Zambito et al. describe an approach for *in vivo* visualization of tumor-associated macrophages to study their role during cancer growth and metastasis. Their imaging agent consists of perfluoro-15-crown-5-ether encapsulated in PLGA-based nanoparticles functionalized *via* PEG chains with mannose and FITC making it suitable as an MRI contrast agent and for *in vitro* optical imaging. Through the functionalization with mannose, the particles have specific targeting properties for mannose-receptor overexpressing cells like tumor-associated macrophages.

The collection of papers in this Research Topic demonstrates the diversity of aspects in imaging technology, and the important role of small animal imaging in developing new imaging techniques and methods. They also highlight challenges still faced in standardization and harmonization of procedures, some specific for the preclinical setting but also still encountered during clinical implementation. To further the tremendous potential of imaging techniques in biomedical research and their use for personalized patient care, standardization and harmonization of methods and protocols are crucial. Hopefully, this Research Topic contributes to an increase in awareness for this need within the research community.

## Author contributions

All authors listed have made a substantial, direct, and intellectual contribution to the work and approved it for publication.

